# Establishment and validation of an eight-gene metabolic–related prognostic signature model for lung adenocarcinoma

**DOI:** 10.18632/aging.202681

**Published:** 2021-02-22

**Authors:** Weishuang Ma, Jiaming Liang, Jinhui Liu, Dongbo Tian, Zisheng Chen

**Affiliations:** 1 Zhouxin Community Health Service, Qingcheng District, Qingyuan, China; 2 State Key Laboratory of Respiratory Disease, The First Affiliated Hospital of Guangzhou Medical University, National Clinical Research Center for Respiratory Disease, Guangzhou, China; 3 The First Affiliated Hospital of Guangzhou Medical University, Guangzhou, China; 4 Department of Respiratory Medicine, The Sixth Affiliated Hospital of Guangzhou Medical University, Qingyuan People’s Hospital, Qingyuan, China

**Keywords:** lung adenocarcinoma, metabolism reprogramming, tumor microenvironment, The Cancer Genome Atlas, prognosis

## Abstract

In this study, we constructed an eight-gene metabolic related signature for LUAD. The eight-gene prognostic signature (including PLAUR, F2, UGT2B17, GNG7, IDO2, ST3GAL6, PIK3CG, and GLS2) exhibited a good prognostic value in the TCGA LUAD training dataset and testing dataset. In addition, the risk score based on the signature model was significantly correlated with immune cell infiltration and expression levels of immune markers in LUAD patients. LUAD cohorts from GEO were used to validate the model, indicating the usefulness of the model. In summary, we developed and validated an eight-gene signature model for LUAD, which can reflect the immune microenvironment characteristics and predict the prognostic outcomes for LUAD patients.

## INTRODUCTION

Globally, lung cancer is the leading cause of cancer-associated mortalities, and lung adenocarcinoma (LUAD) is the most common type of lung cancer accounting for 40% of all cases [1]. In recent years, immune therapy has significantly improved the prognosis of non-small-cell lung carcinoma (NSCLC) patients [2–4]. The use of traditional biomarkers such as tumor mutation burden (TMB), blood TMB, expression levels of PD-1/PD-L1 and circulating tumor DNA (ctDNA) for predicting immunotherapeutic responses is inhibited by several limitations [5]. Therefore, identification of new biomarkers for predicting immune responses and prognosis is urgently needed. There has been a growing awareness on the importance of the tumor microenvironment (TME). The TME is composed of different types of immune cells, extracellular matrix, blood and lymphatic vessels, which exhibit complex interactions with tumor cells and have the ability to influence tumor survival and progression in a beneficial or harmful way [6]. Among the TME components, immune cells are of great importance as they can directly kill the tumor cells, and there are many drug targets which improve or reinvigorate their functions [7].

Studies have documented the importance of metabolic reprogramming in immune cell functions [8]. Tumor metabolism can transform the TME by providing a favorable environment for tumor growth [9]. The transformed TME can enhance or decrease immune cell functions, thereby inhibiting or promoting tumor progression. Metabolic reprogramming in the immune cells inhibits their anti-tumor activities, thereby influencing tumor progress and immunotherapeutic efficacy.

**Figure 1:**
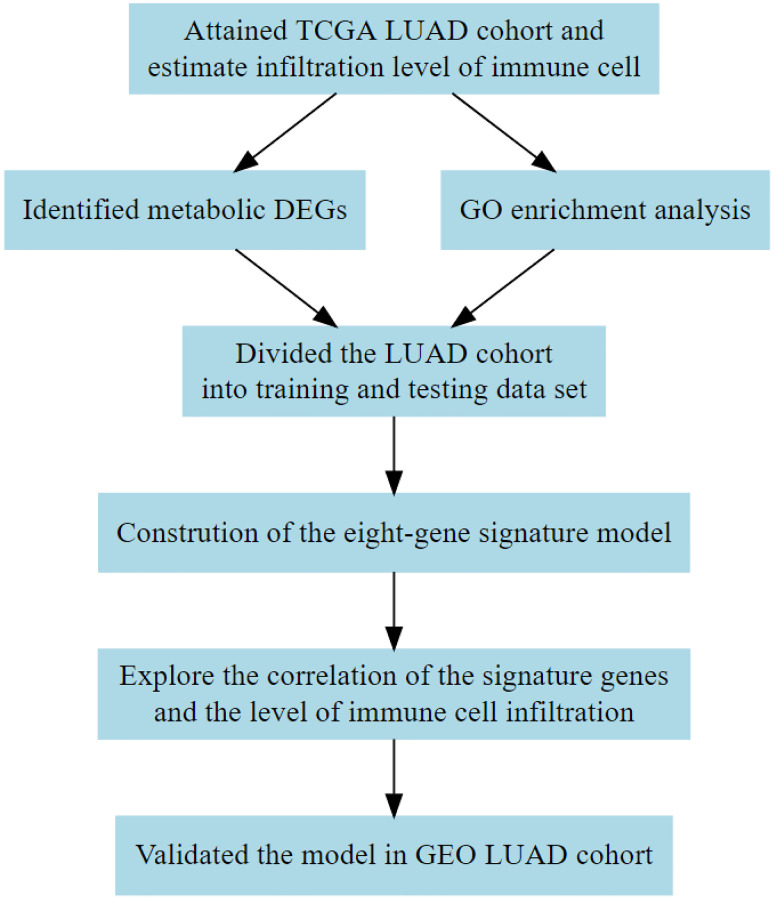
**Flow diagram showing the design of the study.** TCGA, The Cancer Genome Atlas; DEG, differentially expressed gene; GEO, Gene Expression Omnibus; GO, Gene Ontology.

The LUAD is a unique lung cancer subtype with a complex TME [10], and the complex TME can impact on the progression of LUAD. This study evaluated the immune cell and stromal cell landscape of TME in LUAD samples obtained from the TCGA database using algorithms based on the bulk mRNA expression of the tumor samples to determine the prognosis of LUAD. Metabolic associated differentially expressed genes from the two groups were identified based on the median of the estimated score. The relationship between the metabolic genes and the immune cells was then explored. Finally, we identified a metabolic gene set associated with a higher immune cell infiltration that can be used to predict the survival outcomes of LUAD patients. A flow chart of the study design used is shown in Figure 1.

## RESULTS

### Higher estimate score is correlated with better prognosis

**Figure 2:**
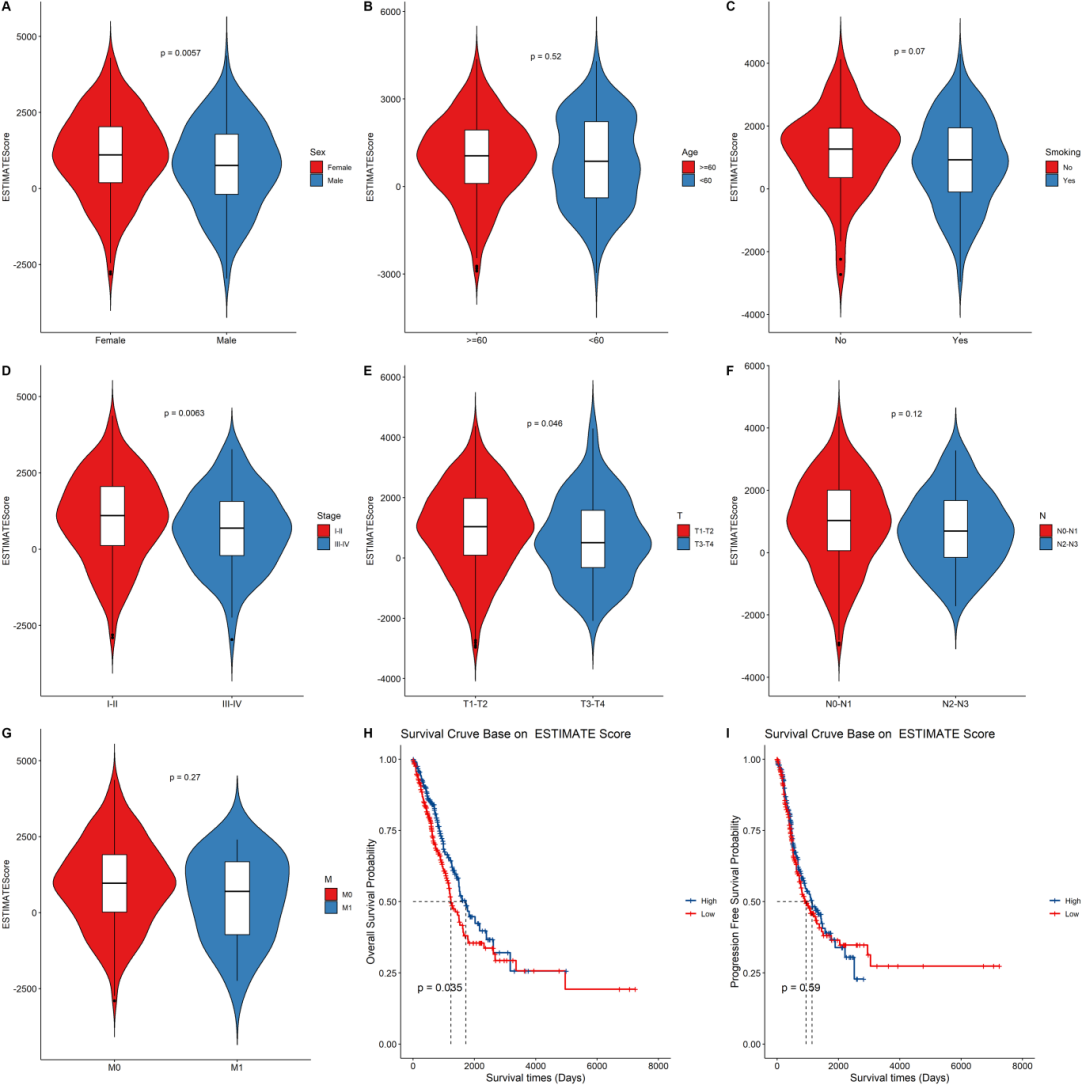
**The relationship between the ESTIMATE score and clinical status, survival outcomes.** (**A–G**) Boxplot showing the difference between the ESTIMATE score and the clinical characteristic. *p* value above the boxplot indicates the difference between the two groups. (**H–I**) The Kaplan–Meier curves for overall survival and progression free survival of LUAD risk groups divided using the median cutoff point of ESTIMATE score.

We first assigned the TCGA LUAD cohort into high and low groups according to their median immune score, stromal score, and estimate score, respectively. Then, we compared the differences in the distribution of clinical characteristics including gender, age, smoking status, TNM stage and survival outcomes between the two groups (Figure 2, Supplementary Figure 1 and Supplementary Figure 2). Clinical characteristics including gender and clinical stage were found to be significantly associated with immune score (Supplementary Figure 1A–1G), stromal score (Supplementary Figure 2A–2G), and estimate score (Figure 2A–2G). The tumor size was significantly associated with the estimate score (Figure 2E). The differences in overall survival time and progression-free survival time were then compared between the two groups. We observed statistically significant differences between the estimate score (Figure 2H) and immune score (Supplementary Figure 1H) and overall survival. However, there were no statistical differences between the estimate score (Figure 2I), stromal score (Supplementary Figure 2I), and immune score (Supplementary Figure 1I) and progression-free survival.

### GO function enrichment analysis of the DEGs

**Figure 3:**
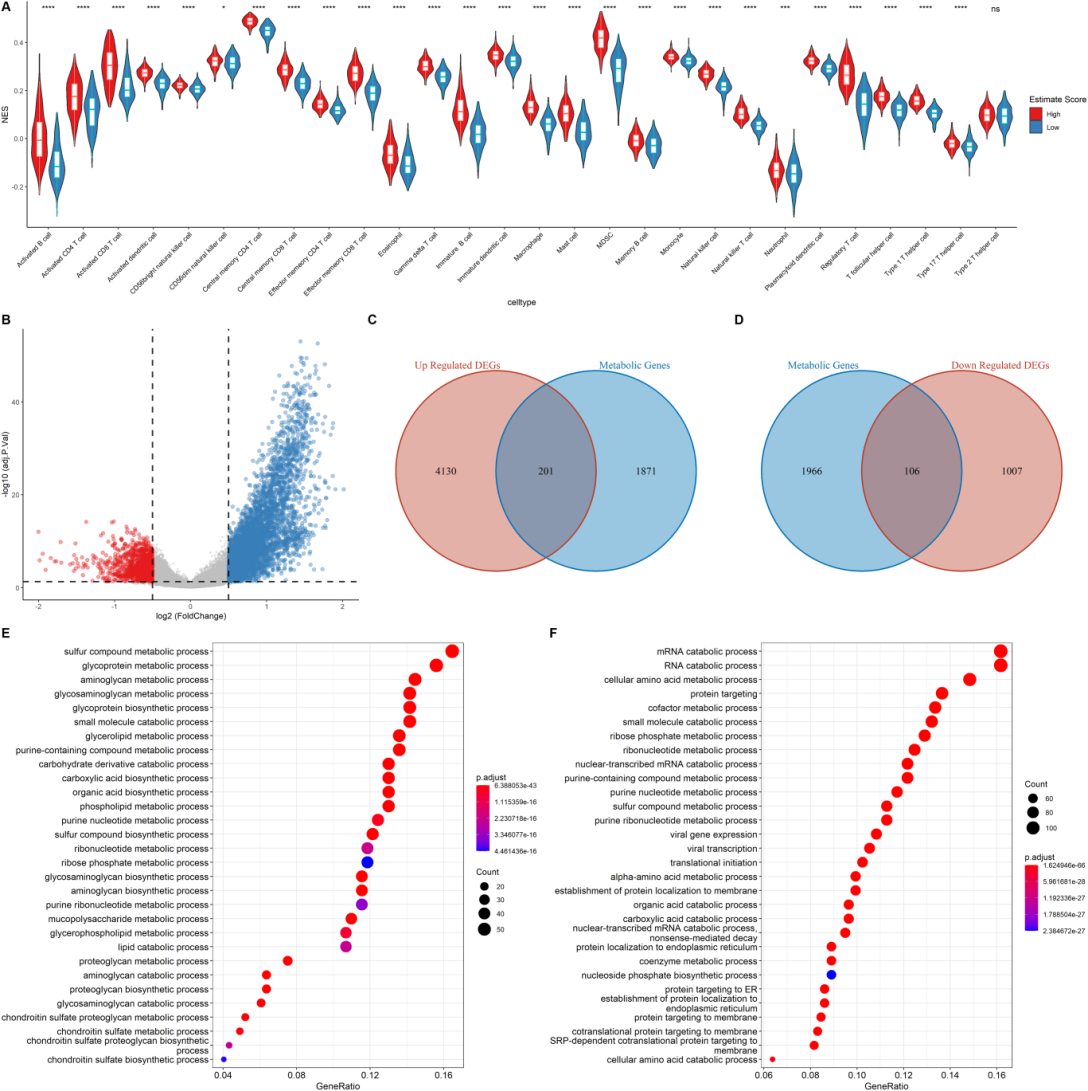
**Functional annotation of the DEGs.** (**A**) Correlation of Immune score and Immune cell score based on the ssGSEA algorithm (**B**) Volcano plot showing the DEGs. The criteria of the DEGs were set as |logFC| > 0.05 and adjusted *p* < 0.05. Red dots and blue dots represent genes that are significantly downregulated or upregulated, respectively. (**C–D**) Venn diagram showing the up regulated and down regulated metabolic DEGs. (**E–F**) GO analysis of up regulated genes and down regulated metabolic genes.

The relative immune cell infiltration level in the TCGA LUAD cohort was estimated using the ssGSEA algorithm. Then, the relative quantity of the 28 immune cells among the two estimate groups were compared. It was found that for most immune cell types, the estimate scores between the two groups were significantly different and the relative quantity of immune cells in the high estimate score group was significantly higher than that in the low estimate score group (Figure 3A). Figure 3A shows that the activated B cell, activated CD4+ T cell, and activated CD8+ T cell exhibited a significant higher score in the high estimate score group than in the lower estimate score group.

Metabolic reprogramming in the tumor environment can impact on the function and population of the infiltrating immune cells [11]. Therefore, the cancer cell metabolic gene set was downloaded from ccmGDB (https://bioinfo.uth.edu/ccmGDB/), and a total of 2,072 metabolic genes were used to determine the association between metabolic gene expression and immune cell infiltration, as well as the relationship between the immune landscape and metabolic genes in the metabolic pathway.

The samples were assigned into high estimate and low estimate groups based on the median of the estimate score. R package limma was then used to determine the differentially expressed genes (DEGs) of the two groups using |logFC| > 0.5 and adjusted *p* < 0.05 criteria. A total of 4,331 up regulated DEGs and 1,113 down regulated DEGs were identified (Figure 3B). As shown in Figure 3C–3D, of the differentially up-regulated and differentially down-regulated genes, 201 and 106 were metabolic-related genes, respectively. GO analysis showed that the upregulated metabolic DEGs were significantly associated with sulfur compound metabolic processes, glycoprotein metabolic processes, and aminoglycan metabolic processes (Figure 3E). However, mRNA catabolic processes, RNA catabolic processes, and cellular amino acid metabolic processes were the enriched pathways among the down-regulated metabolic DEGs (Figure 3F). These findings imply that the two estimate groups exhibited different metabolic phenotypes as well as different infiltration levels of immune cell types.

### Construction of the metabolic gene signature

**Figure 4:**
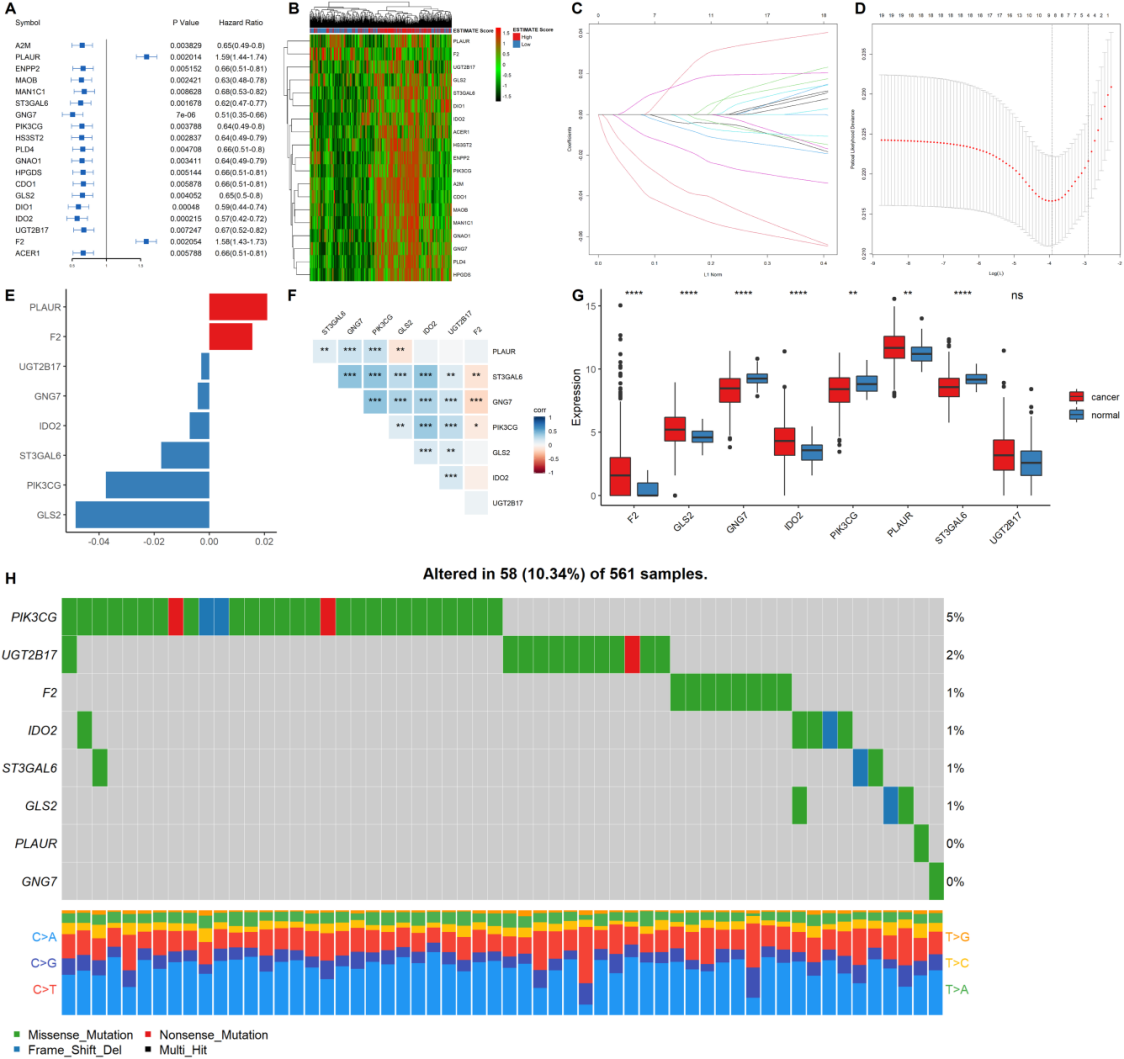
**Construction of a prognostic gene signature.** (**A**) Hazard ratio and p values of the selected candidate genes. (**B**) Heatmap showing the expression profiles of the selected candidate genes. (**C–D**) LASSO cox regression identified eight signature genes that were most correlated with OS. These genes were used to construct a signature model. (**E**) The coefficient value of the selected eight signature genes. (**F**) Expression correlation analysis of the eight signature genes. (**G**) The expression profiles of the eight signature genes in tumor and cancer samples. (**H**) Mutation landscape of the eight signature genes in LUAD.

Univariate COX regression using the survival and gene expression data of LUAD patients was used to evaluate the overall survival prognostic value of the metabolic related DEGs. The 19 most significant genes were selected based on the *p* < 0.01 criteria. The hazard ratio of each gene is shown in the forest plot (Figure 4A). A heatmap representing the expression profiles of the 19 genes is shown in Figure 4B. The dataset was then divided into the training and testing data set at a ratio of 3:1 to construct a prognostic model (training data set *n* = 385; testing data set *n* = 128). LASSO regression was the performed in the training dataset to integrate the roles of these key molecules and to determine the genes that exhibited the greatest importance on the survival outcomes. A signature model of eight genes (PLAUR, F2, UGT2B17, GNG7, IDO2, ST3GAL6, PIK3CG, and GLS2) was constructed based on the LASSO regression results (Figure 4C–4E). The relationships among the relative expression levels of the eight signature genes in the TCGA LUAD cohort were then evaluated. The correlation analysis showed that F2 was significantly negatively correlated with ST3GAL6, GNG7, PIK3CG; GLS2 was significantly negatively correlated with PLAUR, while UGT2B17 was significantly positively correlated with ST3GAL6, GNG7, PIK3CG, GLS2, and IDO2. However, PLAUR did not exhibit any significant correlation with IDO2, UGT2B17, and F2 (Figure 4F). Analysis of this eight-gene signature using the STRING-DB database revealed that GNG7 interacts with F2 and PIK3CG (Supplementary Figure 3A). Next, we evaluated the expression profiles of these signature genes. In the tumor samples, F2, GLS2, IDO2, and PLAUR were shown to be significantly upregulated, while GNG7, PIK3CG, and ST3GAL6 were significantly down-regulated (Figure 4G).

EGFR mutations are highly prevalent in LUAD, especially among East Asian populations. The other main mechanisms of bypass signaling activation include IGF1R, MET, FGFR3, NTRK1, BRAF, ALK, RET, ROS1, and AXL [12]. Then, we elucidated on the correlation between these signature genes and other major drivers or bypass pathways in LUAD. It was found that PLAUR, ST3GAL6, GNG7, PIK3CG, GLS and IDO2 were significantly and positively correlated with most of the oncogenic drivers and bypass signaling, while F2 was negatively correlated with most of them (Supplementary Figure 3B). We also evaluated the mutation landscape of these genes in LUAD. Among the 561 samples, 10.34% of patients exhibited mutations in at least one signature gene. The PIK3CG exhibited the highest mutation frequency followed by UGT2B17, while GNG7 exhibited the fewest mutations in LUAD samples. The waterfall plot presentation of the mutation landscape of the eight signature genes showed that the mutation types were mainly missense mutation (Figure 4H). Most of the eight signature genes were differentially expressed in the tumor and normal tissues of LUAD and exhibited a certain rate of mutation in LUAD.

### Low risk score correlated with better LUAD outcomes

**Figure 5:**
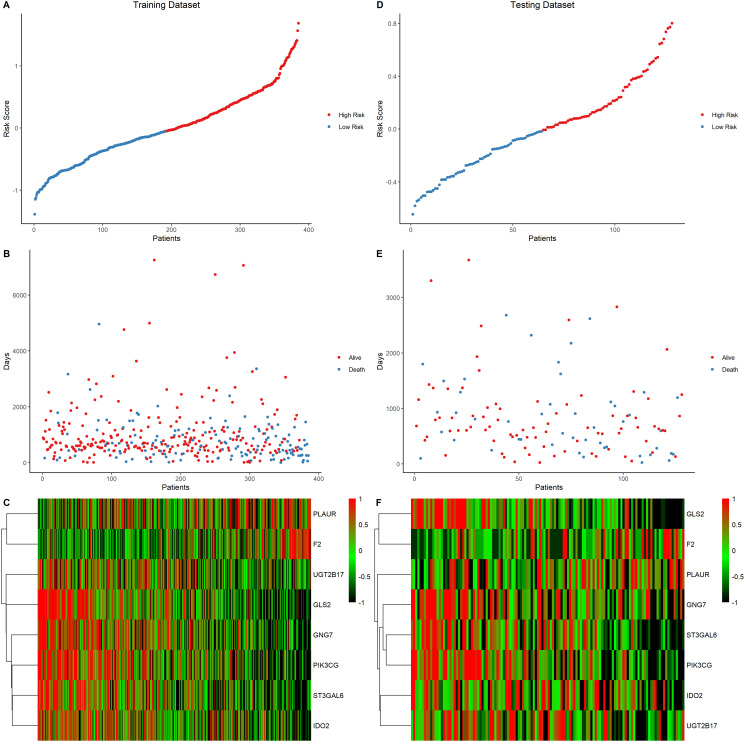
**Eight-gene signature predictor score analysis in training and testing data set.** (**A–C**) Training data set, (**D–F**) Testing data set. The ranked dot plot illustrated the predictor-score distribution of the training data set (A) and testing data set (D). A scatter plot presenting the patients’ overall survival status from training data set (B) and testing data set (E). A heatmap showing the expression profile of the eight signature genes of LUAD patients from training data set (C) and testing data set (F).

The prognostic value of the eight signature genes was evaluated in the training and testing data sets. First, the risk score of each patient was calculated and ranked based on the risk score in the training data set (Figure 5A). The scatter plot was used to present the overall survival status of LUAD patients based on the risk score. Samples in the high-risk group were correlated with a higher mortality rate than those in the low-risk group (Figure 5B). A heatmap presenting the expression profiles of the signature genes showed that tumors with higher risk scores tended to exhibit elevated F2 and PLAUR levels, while those with lower risk scores tended to exhibit elevated UGT2B17, GNG7, IDO2, ST3GAL6, PIK3CG, and GLS2 levels (Figure 5C). This analysis was also performed on the testing dataset which showed consistent results with the training dataset (Figure 5D–5F).

**Figure 6:**
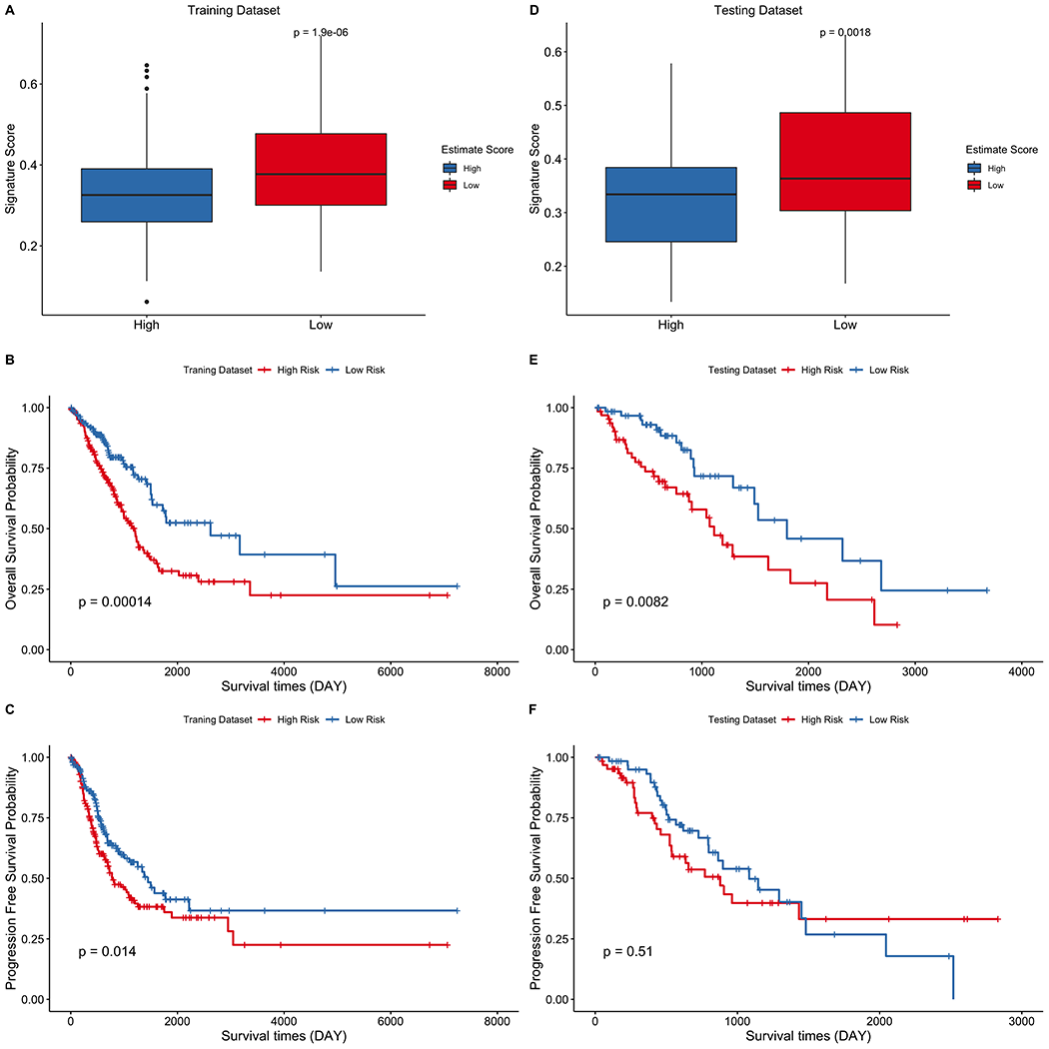
**Eight-gene signature predict survival outcomes in training and testing data sets.** (**A–C**) Training dataset (**D–F**) Testing dataset. LUAD cohort was divided into two groups using the median of estimate score and the risk score of the two groups was then compared. Kaplan–Meier curves for LUAD risk groups divided using the median cutoff point. Patients with higher risk score exhibited significantly poor OS outcomes.

The correlation between the eight-gene signature and the estimate score in LUAD was then evaluated. The risk score was found to be significantly higher in the low estimate score group than in the high estimate score group in both the training (Figure 6A) and testing data sets (Figure 6D). Estimations of the overall survival and progression free survival using the signature score was performed in the training and testing datasets. The low risk score group was correlated with better OS compared to the high-risk score group in both the training (Figure 6B, *p* = 0.00014) and testing datasets (Figure 6E, *p* = 0.0082). However, PFS was significantly different only in the training set (Figure 6C, *p* = 0.014) and not in the testing set (Figure 6F, *p* = 0.51). Collectively, these findings imply that the eight-gene signature model exhibits a good predictive prognostic power in LUAD, and the risk score of the signature model correlated with the estimate score of LUAD.

### The signature score is associated with immune infiltration in LUAD

**Figure 7:**
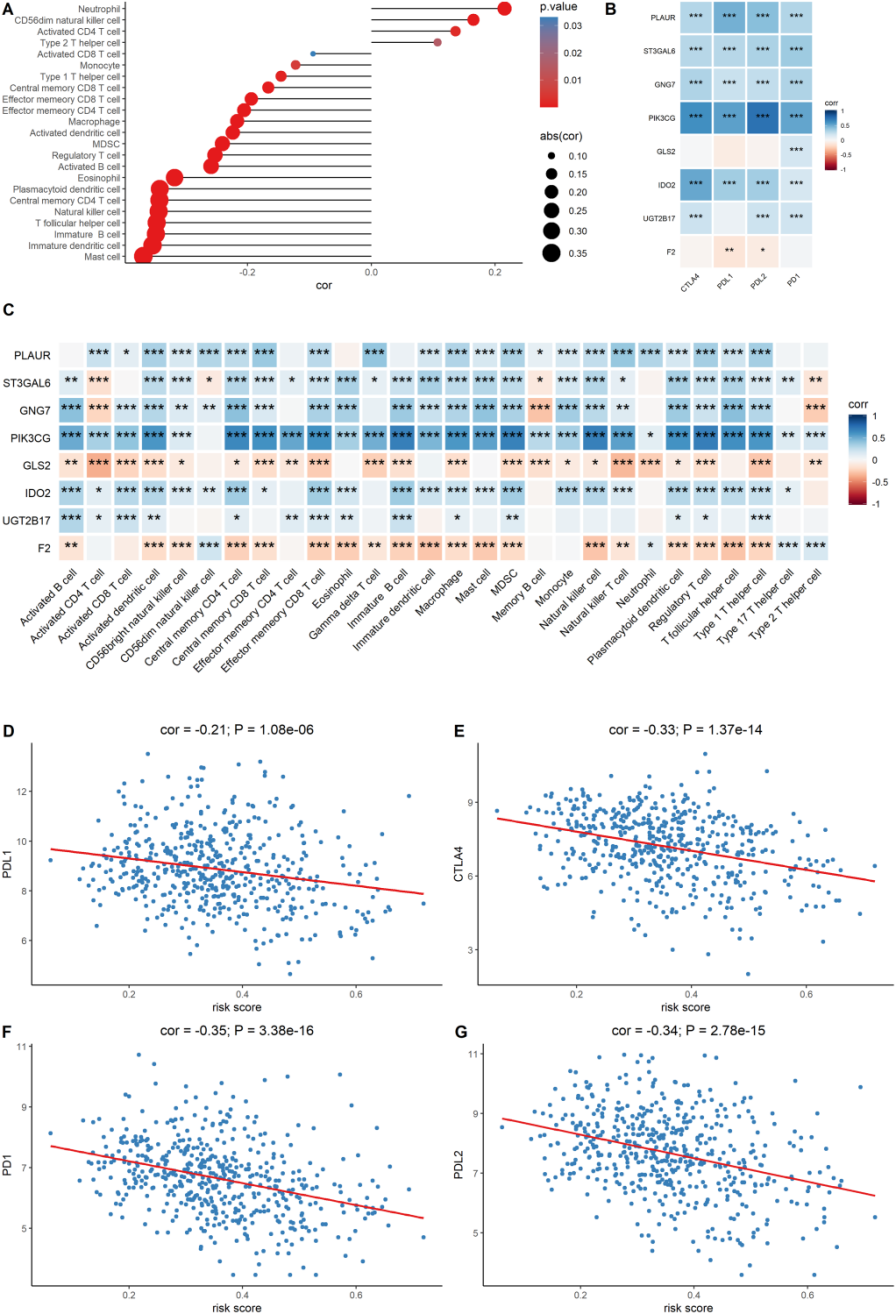
**Evaluation of the correlation between the signature genes and immune characteristics.** (**A**) Correlation between the risk score and immune cell infiltration score. (**B**) Correlation between the expression level of immune markers and the eight signature genes. (**C**) Correlation between each signature gene of the model and each immune cell type. (**D–G**) Correlation between the four immune checkpoint markers and the risk score.

Since the metabolic eight-gene signature correlated with the estimate score, the correlation between the risk score and immune cell scores was then determined. A higher risk score was correlated with a lower abundance of activated CD4+ T cells, natural killer cells, and other immune cell types (Figure 7A). The relationship between the expression of the eight signature genes and the expression of the immune checkpoint molecules was also determined. The expression of PLAUR, GNG7, IDO2, ST3GAL6, and PIK3CG was significantly and positively correlated with the expression of the four checkpoint markers, PD-1, PD-L1, PD-L2, and CTLA-4 (Figure 7B). We also evaluated the correlation between the eight signature genes and immune cell infiltration. The expression levels of PLAUR, UGT2B17, GNG7, IDO2, ST3GAL6, and PIK3CG were significantly and positively correlated with the infiltration of most immune cell types (Figure 7C). Finally, the relationship between risk score and the expression levels of the four immune checkpoint molecules was determined. Risk score was found to be negatively correlated with the expression levels of the four immune checkpoint markers (Figure 7D–7G). Together, these results indicate that the metabolic eight-gene signature is correlated with the expression of immune checkpoint molecules and with the infiltration level of immune cells.

### Verification of the prognostic value of the signature genes using the GEO data set

**Figure 8:**
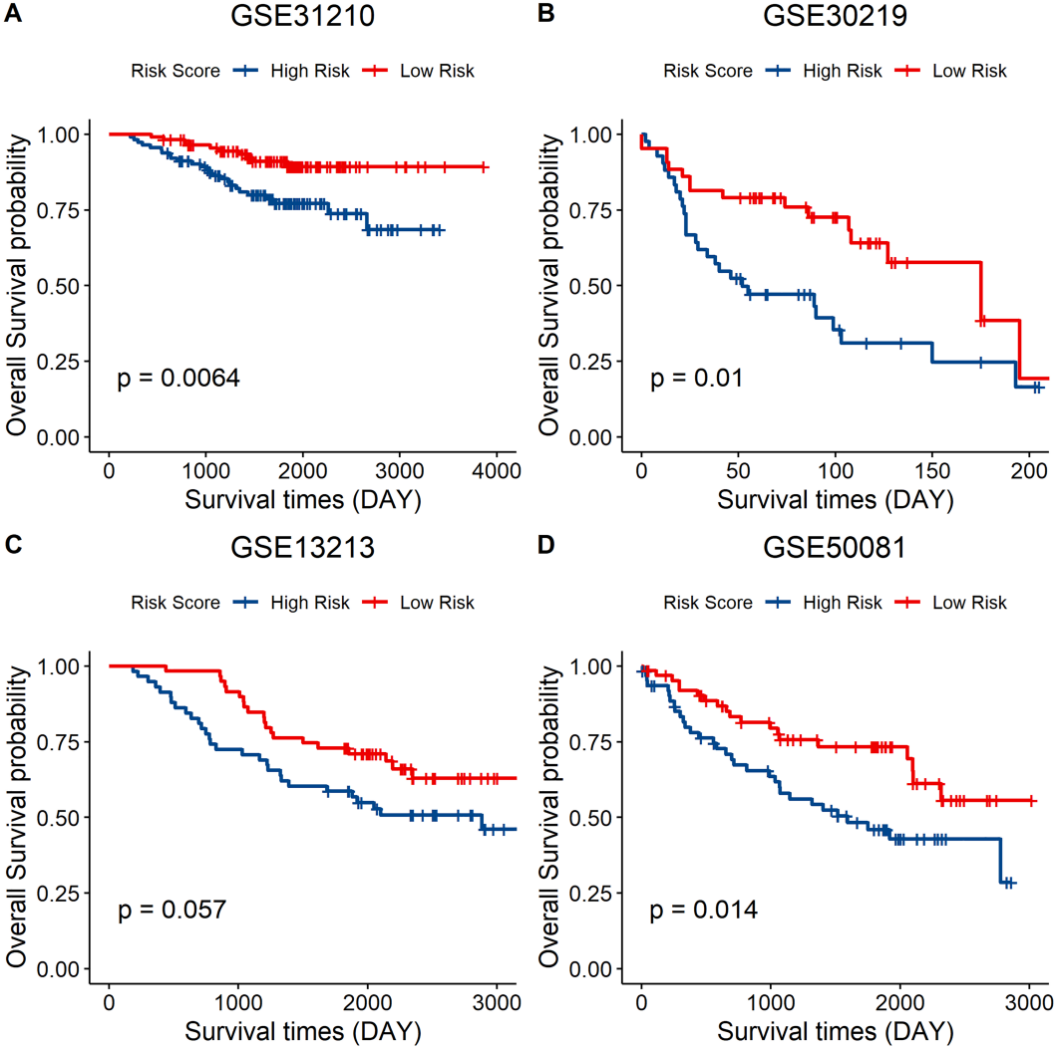
**External validation of the eight-gene signature model.** (**A–D**) Survival curve of GSE31210 (*p* = 0.0064), GSE30219 (*p* = 0.01), GSE13213 (*p* = 0.057), and GSE50081 (*p* = 0.014) indicating that a lower risk score was associated with better overall survival outcomes. Red, low–risk group; blue, high–risk group.

Independent validation of the signature model was performed using the GEO LUAD cohort to verify the ability of the metabolic eight-gene signature model to predict the prognosis. The risk score for each sample of the GEO LUAD cohort was determined using the signature model. The cohort was then divided into two groups based on the median of the risk score. Survival analysis of the four cohorts was then done to validate the prognostic value of the eight gene signature. A lower risk score was correlated with a better overall survival in all the four GEO datasets, including GSE31210 (*p* = 0.0064), GSE30219 (*p* = 0.01), GSE13213 (*p* = 0.057), and GSE50081 (*p* = 0.014) (Figure 8A–8D). Taken together, these results indicate that the eight-gene signature model has a good predictive power for the NSCLC cohort.

## DISCUSSION

The application of RNA-seq and the rapid development of bioinformatics tools have enhanced our understanding of tumors through research. Bianchi et al. constructed a ten-gene predictive model for stage I LUAD [13]; Chen et al. constructed a five-gene signature model for the prediction of relapse-free and overall survival in NSCLC [14]; Shukla et al. constructed a four-gene prognostic signature for LUAD based on TCGA cohort [15]; Boutros et al. constructed a six-gene prognostic signature for NSCLC and validated it using other independent public microarray datasets that include multiple histology types and different stages [16]. However, few of these studies focused on the metabolic genes and their association with TME. Therefore, integrated analysis of metabolic genes in LUAD based on data from GEO and TCGA databases were performed and a metabolic eight-gene signature for predicting the prognosis of LUAD patients was constructed.

We first evaluated LUAD immune infiltration using the TCGA LUAD cohort and identified the differentially expressed metabolic genes between the high estimate score group and the low estimate score group. The data set was then divided into training and testing data set. A prognostic gene signature was then constructed and used to predict the survival outcomes of the LUAD patients using the training data set. The prognostic value of the model was evaluated in the training and testing data sets. A higher risk score was correlated with poor overall survival outcomes. Correlation of the risk scores and immune cells revealed that a lower risk score was associated with increased monocyte infiltration score and T cell infiltration score, which has recently been reported as a predictor for poor prognosis in pancreatic cancer [17]. Immune markers, particularly PD-L1, are used as predictive biomarkers for immunotherapy [18].

We found that the expression levels of immune markers (PD-1, PD-L1, PD-L2, and CTLA-4) are associated with a lower risk score. This implies that the eight-gene signature model could be used to predict immunotherapeutic responses. Finally, we validated the gene signature using a GEO data set. A higher risk score was correlated with poor survival outcomes, which proved the prognostic value of the model (Figure 8).

In this study, we identified the differentially expressed genes between the high estimate score group and the low estimate score group based on the expression profile of LUAD patients. The metabolic related genes were then selected from the DEGs. Nineteen candidate genes were obtained after evaluating the prognostic value of the metabolic DEGs. These genes were then reduced to eight potential predictor genes using the LASSO algorithm. Finally, eight genes (PLAUR, F2, UGT2B17, GNG7, IDO2, ST3GAL6, PIK3CG, and GLS2) were included in the signature model. These signature genes have been reported in previous studies. Some of them function in tumor progression or as prognostic markers for tumor patients.

For example, PIK3CG encodes the phosphatidylinositol-4,5-bisphosphate 3-kinase catalytic subunit gamma isoform (PI3Kγ) enzyme. PI3Kγ is involved in Akt/mTOR signaling, inhibition of NF-κB activation, and in the regulation of tumor immune inhibition by promoting MDSC migration to the tumor environment as well as by stimulating the immunosuppressive transformation of MDSCs [19]. GLS2 is a glutaminase (GLS) isoform, which converts glutamine into glutamate and provides nitrogen for nucleotide and protein synthesis. GLS2 knockdown was shown to inhibit cell proliferation by down-regulating the mTORC1 signaling and inducing autophagy in Gln-dependent lung squamous cell carcinoma cell lines [20]. UGT2B17 is a member of the UDP-glucuronosyltransferases (UGTs) family and is involved in the formation of β-D-glucuronides. The function of UGT2B17 is to maintain androgen homeostasis in the prostate. Elevated UGT2B17 expression levels enhances the progression of castration-resistant prostate cancer by promoting independent androgen receptor signaling and cancer cell mitosis [21]. The cell maintains its cellular homeostasis by autophagy, whereby it consumes its organelles using lysosomes to generate energy for protein synthesis [22]. G protein γ 7 (GNG7) is a heterotrimeric G protein subunit. It has been reported that GNG7 inhibits cell division and induces cell autophagy by inhibiting the mTOR pathway [23]. Moreover, it has been reported that GNG7 is a tumor suppressor gene in clear cell renal cell carcinoma and a lower expression of GNG7 predicts poor overall survival outcomes [24]. ST3GAL6 is a member of the sialyltransferase (STs) family. Overexpression of STs contribute to hypersialylation on the cell surface. Hypersialylation plays an important role in tumor progression. Cancer cells can recruit Siglec-7 and increase sialylated glycans on its surface, thereby protecting it from being killed by natural killer cells [25]. ST3GAL6 expression is significantly suppressed in hepatocellular carcinoma patients [26], and it mediates colorectal cancer progression through the PI3K/Akt signaling [27, 28]. Indoleamine 2,3-dioxygenase 2 (IDO2) is a member of the tryptophan catabolic enzyme. Studies have shown that IDO2 modulates dendritic cell functioning [28], and contributes to immune tolerance by controlling the Treg population [28]. However, the relationship between the IDO2 associated immune response and tumor progression has not been elucidated [29].

The aim of immunotherapy is to reactivate T cells by blocking the interaction of PD-1/PD-L1, thereby, inhibiting PD-1 signaling [30]. In this study, the risk score calculated from the eight-gene signature model was negatively correlated with the expression level of the commonly used immune markers such as PD-L1, PD-1, and CTLA-4, indicating that high risk score patients would not benefit from immune therapy. Correlation analysis showed that the expression level of the eight genes was significantly correlated with the level of immune checkpoint molecules. This finding shows that these genes function in immune responses. PIK3CG is highly expressed in TME and it prevents T cell mediated tumor elimination [31]. Therefore, the eight signature genes may provide clues on the different immunotherapeutic responses. Moreover, a lower risk score was correlated with a higher ssGSEA score of effector memory CD8+ T cell, natural killer cell, and macrophages. This implies that lower risk patients have a higher infiltration of immune cells and, therefore, have a higher probability of benefiting from immune therapy. Therefore, the eight-gene based signature score has the potential for predicting immunotherapeutic responses and LUAD prognosis.

However, this study is associated with several limitations. First, it is a retrospective study. Further prospective studies are required to validate our findings. Secondly, the study did not verify the ability of the eight genes signature to predict immune responses in LUAD patients because we lacked the clinical data on patients receiving immunotherapy. Finally, other patient characteristics such as age, tumor size, and lymph node status were not included in our prognostic analysis.

## MATERIALS AND METHODS

### Data collection and pre-processing

Clinical data, TCGA RNA-seq data, and probe annotation files of the LUAD patients were downloaded from the UCSC xena browser (https://xenabrowser.net/) and used to obtain the gene expression profiles of the human LUAD patients. Data for the normal tissue was discarded while samples with no clinical data were excluded. Finally, a total of 513 tumor samples were retained and the TCGA LUAD cohort was randomly divided into training and testing data set at a ratio of 3:1 (training data set *n* = 385; testing data set *n* = 128). The two groups exhibited a similar estimate score distribution and other clinical characteristics. GSE31210, GSE30219, GSE13213, and GSE50081 datasets were downloaded from Gene Expression Omnibus (GEO) database in R using R package “GEOquery” [32]. The probe IDs were then transformed into gene symbols according to the annotation files, and the cancer metabolism gene set was obtained from ccmGDB (https://bioinfo.uth.edu/ccmGDB/).

### Evaluation of the immune score and stromal score

The R package “ESTIMATE” was used to infer the fraction of immune cells and stromal cells in the patient's tumor samples [33]. The ESTIMATE algorithm was designed to calculate the immune and stromal scores of each sample based on the expression of certain stromal cell and immune cell genes. The results obtained from the ESTIMATE algorithm are presented in three categories; where immune score represents the score of immune cell infiltration, stromal score represents stromal cell infiltration, and estimate score represents the sum of both the immune score and stromal score. The LUAD samples were divided into two groups based on the expression level of the estimate score of each sample.

### Determination of the infiltration level of immune cells in LUAD

The R package “GSVA” uses a method called Gene Set Variation Analysis (GSVA), which implements a non-parametric unsupervised method to assess the underlying pathway activity using gene expression microarray and RNA-seq data. By applying this approach, we can use the traditional analytical methods such as correlation analysis and survival analysis in a pathway focused manner. A set of immune cells' gene marker, consisting of 782 genes representing 28 immune cell types from innate and adaptive immunity, was obtained from a previous study and used to evaluate the infiltration of different immune cell types in the tumor microenvironment [34]. The immune cell types included dendritic cells, B cells, NK cells, MDSC, neutrophils, T cells, among others. Subsequently, the ssGSEA algorithm from R package “GSVA” was used to determine the infiltration level of each immune cell type in LUAD using the expression profiles [35]. Each sample was evaluated using the gene signature expressed by the immune cell and the calculation was then performed using the ssGSEA algorithm.

### Identification of Differentially Expressed Genes (DEGs) and functional enrichment analysis

The LUAD cohort was divided into two groups based on the median of the estimate score. R package “limma” was used to determine the DEGs between the two groups [36]. Adjusted *p* < 0.05 and |logFC| > 0.5 were set as the cutoff criteria to determine the significantly differentially expressed genes. The gene Ontology system of classification was used to classify genes into different gene sets based on their functions. The genes were then assigned with their GO terms. Gene Ontology (GO) term enrichment can be used to interpret which GO terms are over-expressed or under-expressed when given a set of up-regulated or down-regulated genes. The metabolic genes with a criteria of adjusted *p* < 0.05 were used to explore the metabolic landscape between the two groups. The metabolic genes were then used in the GO analysis using R package “ClusterProfiler” to identify the enriched biological pathways [37]. A cutoff value of 0.05 was set to obtain significant results for biological process (BP), cellular components (CC), and molecular functions (MF). R package “ClusterProfiler” was then used to visualize the GO enrichment results.

### Generation of a prognostic model using LASSO Regularization to evaluate the mutation profile of the signature genes

Least absolute shrinkage and selection operator (LASSO) is a type of linear regression. Adding a penalty equal to the absolute value of the magnitude of some coefficients can result in the coefficients becoming zero, and thus they can be removed from the model. Therefore, a model with few coefficients can then be created. The candidate gene expression profiles were obtained from the training data set (*n* = 385) and R package “glmnet” was used to perform LASSO regularization to reduce the coefficients. This was followed by selection of the most robust markers to construct the risk score signature, which included eight genes. The following formula was used:

Risk Score = 0.021 × PLAUR – 0.017 × ST3GAL6 – 0.004 × GNG7 – 0.038 × PIK3CG – 0.048 × GLS2 – 0.007 ×IDO2 – 0.004 × UGT2B17 + 0.016 × F2.

“Maftools” was selected for mutation analysis based on the model constructed by LASSO regularization to explore the mutation frequency of the signature genes in LUAD [38].

### Testing data set validation

The expression profiles of the signature genes were extracted from the testing data set, then used in the prognosis model for calculation. The predicted risk score was calculated and its association with survival outcomes was further analyzed.

### Survival analysis

Univariate Cox proportional hazards regression analysis was used to evaluate the association between the expression level of the metabolic DEGs and the overall survival (OS) of LUAD patients. Metabolic DEGs with *p* < 0.05 based on the log-rank test were selected as candidate genes for construction of the prognosis model. The risk score for each sample was calculated based on the signature model to evaluate the association between the gene signature and the prognosis of LUAD patients. The samples were classified into either high risk or low risk groups depending on the median risk score. Kaplan-Meier curve and log-rank test were used to compare the differences in overall survival and progression free survival outcomes between the predicted high risk and low risk groups. *p* ≤ 0.05 was set as the significant level. All the survival analyses and log-rank tests were performed using R package survival, while the R package “surviminer” was used to plot the Kaplan-Meier curve.

### Statistical analysis

Univariate analysis of survival outcomes was performed using the log-rank test. The correlation relationships between the risk score and immune markers, the risk score and signature gene expression, the signature gene expression and immune cell infiltration score, and the risk score and immune cell infiltration score were determined by Pearson correlation. A two-tailed student t-test was used to compare the two groups. *p* ≤ 0.05 was set as the threshold for statistical significance. All statistical analyses were performed in R version 4.0.2.

### Data availability statement

The data that supports this study is available in GSE31210, GSE30219, GSE13213, and GSE50081 at https://www.ncbi.nlm.nih.gov/gds. TCGA RNA-seq and clinical data of the LUAD patients was downloaded from the UCSCxena browser (https://xenabrowser.net/), while the cancer cell metabolism gene set was downloaded from ccmGDB (https://bioinfo.uth.edu/ccmGDB/).

## Supplementary Material

Supplementary Figures
