# Supplementary material for: Establishment and validation of an eight-gene metabolic–related prognostic signature model for lung adenocarcinoma
Source: Aging (Albany NY). 2021 Feb 22;13(6):8688–705. doi: 10.18632/aging.202681 (PMC8034925; doi:10.18632/aging.202681)
Supplement: Supplementary Figures [file aging-13-202681-s001.pdf]

SUPPLEMENTARY FIGURES

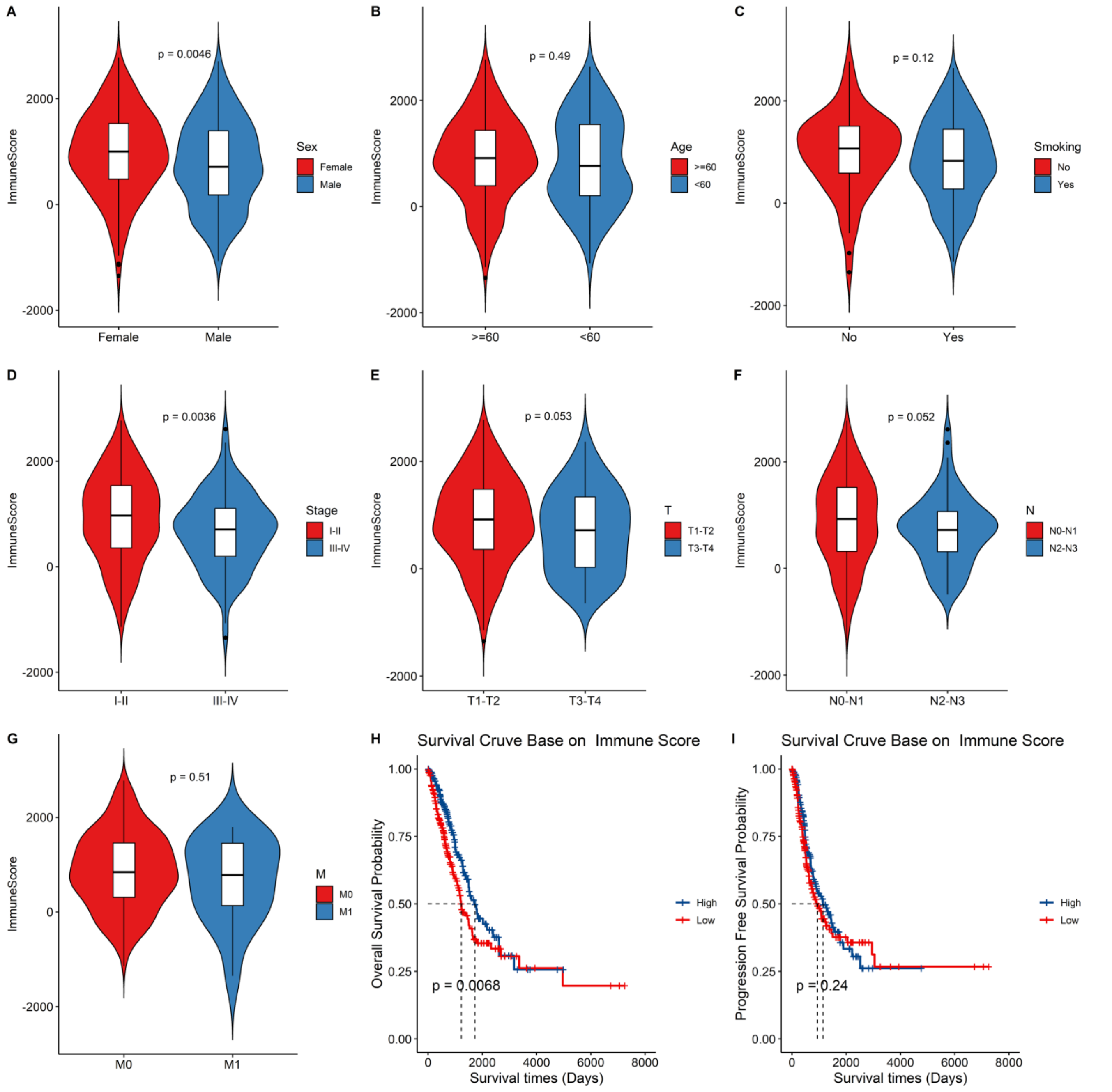

Supplementary Figure 1. Relationship between the Immune score and clinical status, survival outcomes. (**A**–**G**) Boxplot represent the difference between the immune score and clinical characteristics. *p* value above the boxplot indicates the difference between the two groups. (**H**–**I**) The Kaplan–Meier curves for overall survival and progression free survival of LUAD risk groups divided using the median cutoff point of immune score.

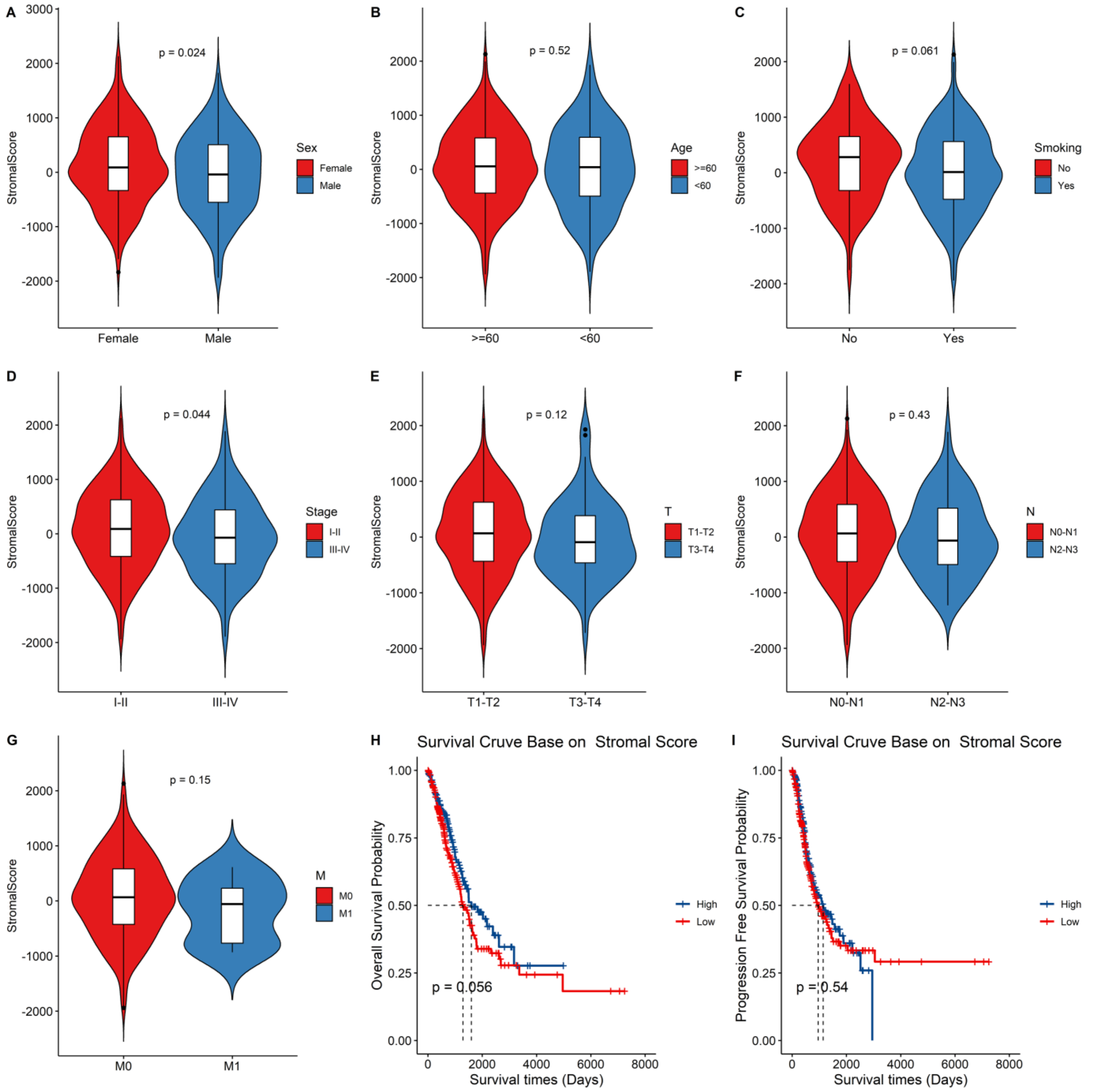

Supplementary Figure 2. Relationship between the Stromal score and clinical status, survival outcome. (**A**–**G**) Boxplot representing the difference between the stromal score and the clinical characteristic. *p* value above the boxplot indicates the difference between the two groups. (**H**–**I**) The Kaplan–Meier curves for overall survival and progression free survival of LUAD risk groups divided using the median cutoff point of Stromal score.

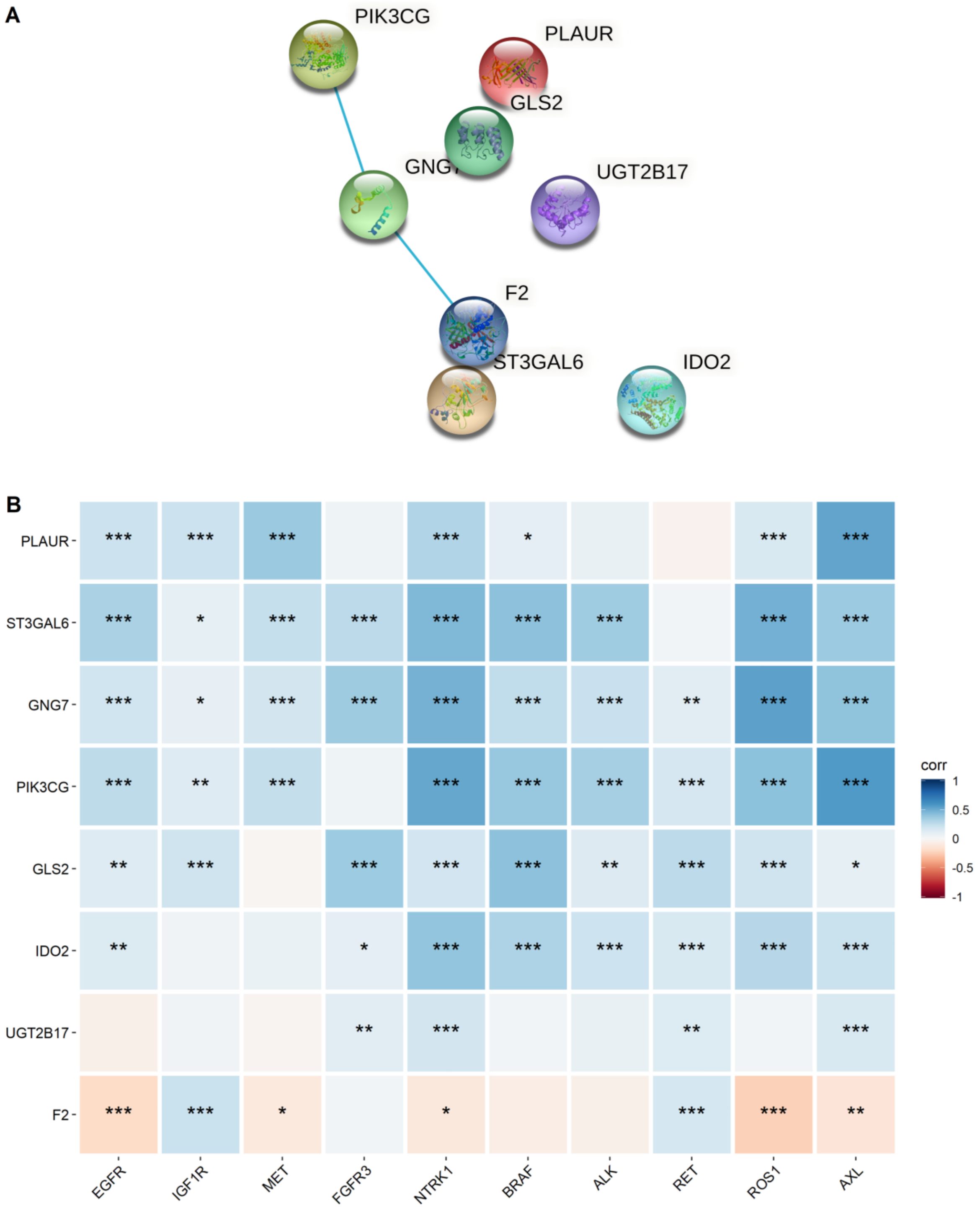

Supplementary Figure 3. (**A**) Analysis of the interactions among the eight signature genes using the STRING-DB database. (**B**) Correlation analysis of expression of the eight signature genes and LUAD oncogenic drivers and bypass signaling.
